# A Survey of Transitions of Young People From Child & Adolescent Mental Health Services (CAMHS) to Adult Mental Health Services (AMHS)

**DOI:** 10.1192/bjo.2022.454

**Published:** 2022-06-20

**Authors:** Umama Khan, Jeremy Turk

**Affiliations:** Isle of Wight NHS Trust, Isle of Wight, United Kingdom

## Abstract

**Aims:**

To evaluate current transition service provision from CAMHS to AMHS focusing on information sharing, transition planning and continuity of care. Many young people find service transition from CAMHS to AMHS stressful, sometimes because of there being multiple simultaneous transitions from child centred services to adult oriented models of care. If not handled well, this can lead to drop out from services, repeated assessments, suboptimal treatment and support, and raised likelihood of emergency psychiatric admissions. Longitudinal planning and sensitive management of transition is vital, because of transition being a risk period for young people.

**Methods:**

A retrospective case note survey of service users who had transitioned from 1st February 2020 to 31st January to 2022 was undertaken. Fifteen individuals transitioned: 9 females and 6 males. All were White British except for one who was Sudanese and an asylum seeker. Six out of 15 young people had a diagnosis of Autism Spectrum Disorder (ASD) in addition to other diagnoses making transition more difficult, as more agencies were involved requiring multiple meetings prior to transition to adult services. One case of emerging personality disorder, despite our best efforts for smooth transitioning, had already disengaged from CAMHS.

**Results:**

Most individuals transitioned successfully to adult mental health services. Two were transferred to learning disability, and one to early intervention in psychosis services, the diagnoses having been confirmed by the treating psychiatrist near the patient's 18th birthday. One was transferred to a rehabilitation service. Only one referral was declined.

The transition pathway is patient centred, and provides clear transition plans to young person, family and carers. In the past 4 months AMHS, because of lack of resources, have not been able to identify a named worker till a few weeks before the patient's 18th birthday, which conflicts with NICE guidelines.

**Conclusion:**

An effective transition service was found to include

- A designated psychiatrist to facilitate smooth transition of complex cases to AMHS, providing continuity of care, good intra and inter agency working and maximising patient welfare.
- CAMHS preparation of young persons for transition commencing six months before their 18th birthday.
- Prior researching of best transition destinations.
- An overlap period of CAMHS/AMHS joint working.
- Identified CAMHS and AMHS transition coordinators.
- Patient and family engagement with process.
- Mindfulness and awareness of AMHS eligibility criteria.

